# Supplementary material for: The Governance of Childhood Vaccination Services in Crisis Settings: A Scoping Review
Source: Vaccines (Basel). 2023 Dec 14;11(12):1853. doi: 10.3390/vaccines11121853 (PMC10747651; doi:10.3390/vaccines11121853)
Supplement: Supplementary file 1 [file vaccines-11-01853-s001.zip › Supplementary material S1_The governance of childhood vaccination services in crisis settings.pdf]

The governance of childhood vaccination services in crisis settings: a scoping review

Supplementary material S1
Countries with a consolidated appeal/humanitarian response plan during 2010 – 2021

1. Afghanistan
2. Bangladesh
3. Benin
4. Burkina Faso
5. Burundi
6. Cameroon
7. Central African Republic
8. Chad
9. Colombia
10. Côte d'Ivoire
11. Democratic Republic of the Congo
12. Djibouti
13. El Salvador
14. Ethiopia
15. (The) Gambia
16. Guatemala
17. Haiti
18. Honduras
19. Iraq
20. Kenya
21. Lebanon
22. Liberia
23. Libya
24. Mali
25. Mozambique
26. Myanmar
27. Niger
28. Nigeria
29. Pakistan

30. Palestine
31. Philippines
32. Somalia
33. South Sudan
34. Swaziland/Eswatini
35. Sudan
36. Syria
37. Uganda
38. Ukraine
39. Venezuela
40. Yemen
41. Zimbabwe
