# Supplementary material for: The Governance of Childhood Vaccination Services in Crisis Settings: A Scoping Review
Source: Vaccines (Basel). 2023 Dec 14;11(12):1853. doi: 10.3390/vaccines11121853 (PMC10747651; doi:10.3390/vaccines11121853)
Supplement: Supplementary file 1 [file vaccines-11-01853-s001.zip › Supplementary material S2_The governance of childhood vaccination services in crisis settings.pdf]

The governance of childhood vaccination services in crisis settings: a scoping review

Supplementary material S2

Search terms

**Concept**	**Scientific journal database search (Terms for title and abstract search)**	**Grey literature search**
Countries with a consolidated appeal/humanitarian response plan during 2010 – 2021	[bangladesh* or colombia* or (“salvador*” or “guanaco*”) or guatemala* or hondura* or (“philippine*” or “filipin*”) or somali* or ukrain* or “central africa*” or chad or chadian or "south* sudan*" or haiti* or afghan* or niger* or congo* or mali or malian or benin* or gambia* or liberia* or burkina* or yemen* or mozambi* or nigeria* or ("cote d'ivoire*" or "ivory coast*" or "ivorian*") or sudan* or djibouti* or pakistan* or zimbabwe* or burundi* or ("eswatini*" or "swazi*" or "swati*") or uganda* or ethiopia* or kenya* or cameroon* or ("myanma*" or "burm*") or ("palestin*" or "west bank*" or "gaza*") or iraq* or leban* or libya* or syria* or venezuela*]<br><br>A subject heading search was not conducted for this concept	“Low- and middle-income countries”, Humanitarian, Crisis, Fragile
Vaccination	[Vaccin*, immunis*, immuniz*]<br><br>A subject heading search was also conducted for this concept	Vaccination, immunisation, immunization
Governance	[governi*, governa*, governe* leadership, accountab*, stewardship, decision-making]<br><br>A subject heading search was also conducted for this concept	Governance, leadership, accountability, decision-making
Service Planning	[service*] adj2 [plan*, develop*]<br><br>OR<br><br>[fund*, financ*, spend*, subsid*]<br><br>OR<br><br>[purchas*, procur*]<br><br>A subject heading search was also conducted for this concept	Service planning, Finance, Procurement
