# Supplementary material for: The Governance of Childhood Vaccination Services in Crisis Settings: A Scoping Review
Source: Vaccines (Basel). 2023 Dec 14;11(12):1853. doi: 10.3390/vaccines11121853 (PMC10747651; doi:10.3390/vaccines11121853)
Supplement: Supplementary file 1 [file vaccines-11-01853-s001.zip › Supplementary material S3_The governance of childhood vaccination services in crisis settings_revised 13Dec23.pdf]

Supplementary material S3

Definitions and scope of analytical tools derived from the Governance Analysis Framework

Source: Hufty, M. Investigating Policy Processes: The Governance Analytical Framework (GAF). In Research for Sustainable Development: Foundations, Experiences, and Perspectives; Weismann, Urs, Hurni, Hans, Eds.; Geographica Bernensia: Bern, Switzerland, 2011; pp. 403–424.

Analytical tool	Definition/Scope
**Defining the problem**	- What constitutes a 'problem'? It is whatever actors consider as "issues at stake". GAF acknowledges that the problem may be completely different for each actor, and assumes that the "problem" is the subject of a governance process<br>- The analysis should convert what the actors present as a problem into a sociological question – the ability to do that depends greatly on the context, methodology, techniques and experience of the research.
**Understanding social norms**	- What are social norms? They are what guide, prescribe, and sanction collective and individual behaviour. Essentially, they are the 'rules of the game', and the rules that determine how the rules of the game are established.<br>- Norms are based on values or beliefs (the sense of what is right is right or wrong) and include prescription (what one should or should not do). When norms recur, they are institutionalised.<br>- They can be legal (inscribed in law), formal (recognized by the strategic actors) or informal (created by the practices of the actors).<br>- They may be formulated at various levels and transferred to other actors
**Differentiating actors**	- Who are considered actors? All the stakeholders (individuals or groups), whether "formal or informal" (recognised by strategic actors or not).<br>- Characterise the actors: category, mandates/missions, modes of expression and action, vaccination roles, formal and real responsibilities or functions covered, space of intervention, position on vaccination governance<br>- Determine the relative power of actors: assess the resources controlled (vaccine stock and supplies, funding, cultural, symbolic, etc.); the will and capacity to mobilise these resources; analyse the contexts of effective mobilisation for vaccinations interventions; analyse if/how they interact with other actors (reciprocal, negotiating, directive interactions).<br>-
**Investigating nodal points**	- What are nodal points? These are the physical or virtual spaces where various problems, actors, and processes converge.<br>- Identify the different nodal points.<br>- Characterise the nodal points: the relationships and their effects on the vaccination decisions, and whether they are favourable or unfavourable to a process of change
**Analysing processes**	- What are processes? Processes are successions of states through which a system passes.<br>- The analysis aims to identify sequences which allow us to evaluate the direction in which these processes are evolving (over time) and to locate factors which favour change (interactions between actors and their relation with the changes of the norms observed)
